# Highly Sensitive Plasmonic Detection of the Pancreatic Cancer Biomarker CA 19-9

**DOI:** 10.1038/s41598-017-14688-z

**Published:** 2017-10-30

**Authors:** Zaynab A. R. Jawad, Ioannis G. Theodorou, Long R. Jiao, Fang Xie

**Affiliations:** 1 Department of Materials and London Centre for Nanotechnology, Imperial College London, SW7 2AZ London, UK; 2 Department of Cancer and Surgery, Imperial College London, SW12 0HS London, UK

## Abstract

Plasmonic gold (Au) nanotriangular arrays, functionalized with a near infrared (NIR) fluorophore-conjugated immunoassay to Carbohydrate Antigen 19-9 (CA 19-9), a pancreatic cancer biomarker, produce optically tunable substrates with two orders of magnitude fluorescence enhancement. Through nanoscale morphological control, the sensitivities of the plasmonic nanotriangular arrays are controllable, paving the way of such optical platforms for multiplexing. Here, we report a limit of detection (LOD) of 7.7 × 10^−7^ U.mL^−1^ for CA 19–9 by using such tunable Au nanotriangular arrays, a great improvement compared to commercially available CA 19–9 immunoassays. The linear dynamic range was from 1 × 10^−6^ U.mL^−1^ to 1 U.mL^−1^, *i.e*. up to six orders of magnitude. Moreover, high specificity was demonstrated, together with successful validation in serum samples. Their superior tunable sensitivity, along with efforts to combine CA 19–9 with other biomarkers for improved accuracy, open up the possibility for multiplexed NIR-fluorescence enhancement microarrays, for early cancer diagnosis and therapeutic monitoring.

## Introduction

Only 8% of pancreatic cancer patients survive longer than 5 years after diagnosis^1^, mainly due to the poor detection of the disease at an early stage. Patients only start to show symptoms in the late stages^2^, and only 15% of cases are suitable to undergo surgery at the point of diagnosis^3,4^. To improve survival rates, the development of a non-invasive screening test, would be extremely beneficial^5^. This test would allow the identification of specific biomarkers at the early stage, when tumors are symptom-free and non-metastatic.

Analytical protein microarrays allow for detailed analysis of low sample volumes^6,7^, making them a potentially useful tool for cancer screening. In pancreatic cancer diagnosis, in particular, a variety of associated body fluids may be sampled and tested (*e.g*. bile and pancreatic cyst fluids), which are only obtained in small quantities^8^. The expression of a variety of potential candidate biomarkers has already been identified in these fluids for diagnosis^9,10^. When used in combination, these biomarkers provide dramatically improved specificity. Multiplexed protein microarrays would therefore make significantly more specific diagnostic platforms for detection. Unfortunately, current state-of-the-art protein microarrays suffer from poor detection limits^11,12^. Various methods have been proposed to improve the sensitivity and detection limits of protein microarray assays^13–15^, based, for example, on enzymatic amplification or carbon nanotube Raman tags^16,17^.

Metal enhanced fluorescence (MEF) has emerged as a promising strategy, as it can dramatically enhance the assay sensitivity, without complicating the assay with additional preparation steps or requiring specialized measurement equipment^18,19^. This approach takes advantage of the amplification of fluorescence emission intensity of fluorophores located proximal to plasmonic nanostructures, to produce higher signal-to-noise ratios. By selecting fluorophores emitting in the near infrared range (NIR, 700–900 nm), interference from biomolecule auto-fluorescence is also minimized^20,21^. However, the plasmonic microarrays used in previous work, for the detection of bowel cancer biomarker, carcinoembryonic antigen (CEA) and three lung cancer biomarkers^22,23^, lacked systematic control of their nanoscale structure and the corresponding optical properties. Thus, no control over the resulting fluorescence enhancement factors was achieved, the key step for maximizing the sensitivity and tuning the sensitivities of multiplexed microarrays to the abundances of different biomarkers of interest present in a single sample.

**Figure 1:**
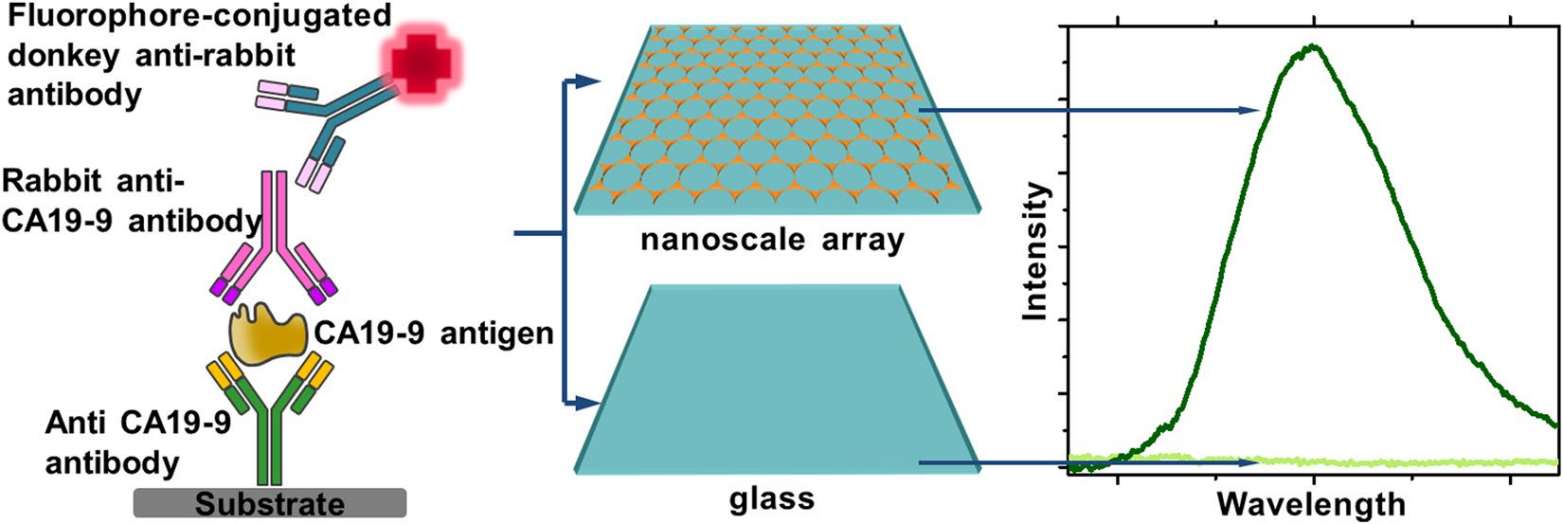
Near-infrared fluorescence enhancement immunoassay for the quantification of the pancreatic cancer biomarker CA 19–9, demonstrates superior sensitivity compared to glass controls. Schematic diagram of the CA 19–9 “sandwich” protein microarray performed on Au-nanotriangular substrates, resulting to up to two orders of magnitude fluorescence intensity enhancement compared to glass controls.

In the present work, protein microarrays based on NIR plasmonic gold nanostructures are reported, as illustrated schematically in Fig. 1. Nanoscale control of the plasmonic substrate structure by colloidal lithography, with advantages of large area and low cost, enabled tuning of their optical response, consequently allowing their sensitivities to be controllable. Previous studies have demonstrated highly sensitive detection of various protein analytes using metal nanotriangles produced by nanolithography, a technique developed by Van Duyne *et al*.^24^. These biosensors use alternative plasmonic effects such as surface-enhanced Raman spectroscopy (SERS) or surface plasmon resonance (SPR) sensing^24,25^. Here, using the fluorescence enhanced plasmonic protein arrays, NIR fluorescence enhancement of almost two orders of magnitude was measured. The arrays were also successfully tested in serum samples spiked with CA 19–9, and validated against a fluorescent immunoassay. CA 19–9 is the only validated biomarker for pancreatic cancer^26^, and was used as proof-of-concept .Unfortunately, the serum levels of CA 19–9 may be elevated in conditions other than pancreatic cancer, making the identification of novel biomarkers for early diagnosis of PC urgently required^27^. Multiple investigations have combined CA19–9 with other biomarkers in order to improve the accuracy of diagnosis^27^, further highlighting the importance of a multiplexing strategy. The nanoscale controllable sensitivities of the plasmonic nanotriangular arrays presented here pave the way for such multiplexed platforms. Along with their compatibility with standard microarray scanners, these novel NIR-MEF protein microarrays are directly translatable to other biomarkers and open up new possibilities for the early diagnosis and monitoring of pancreatic cancer, by combining the advantages of MEF and NIR.

## Results and Discussion

### Au nanotriangular arrays with nanoscale controlled structure fabricated by colloidal lithography

**Figure 2:**
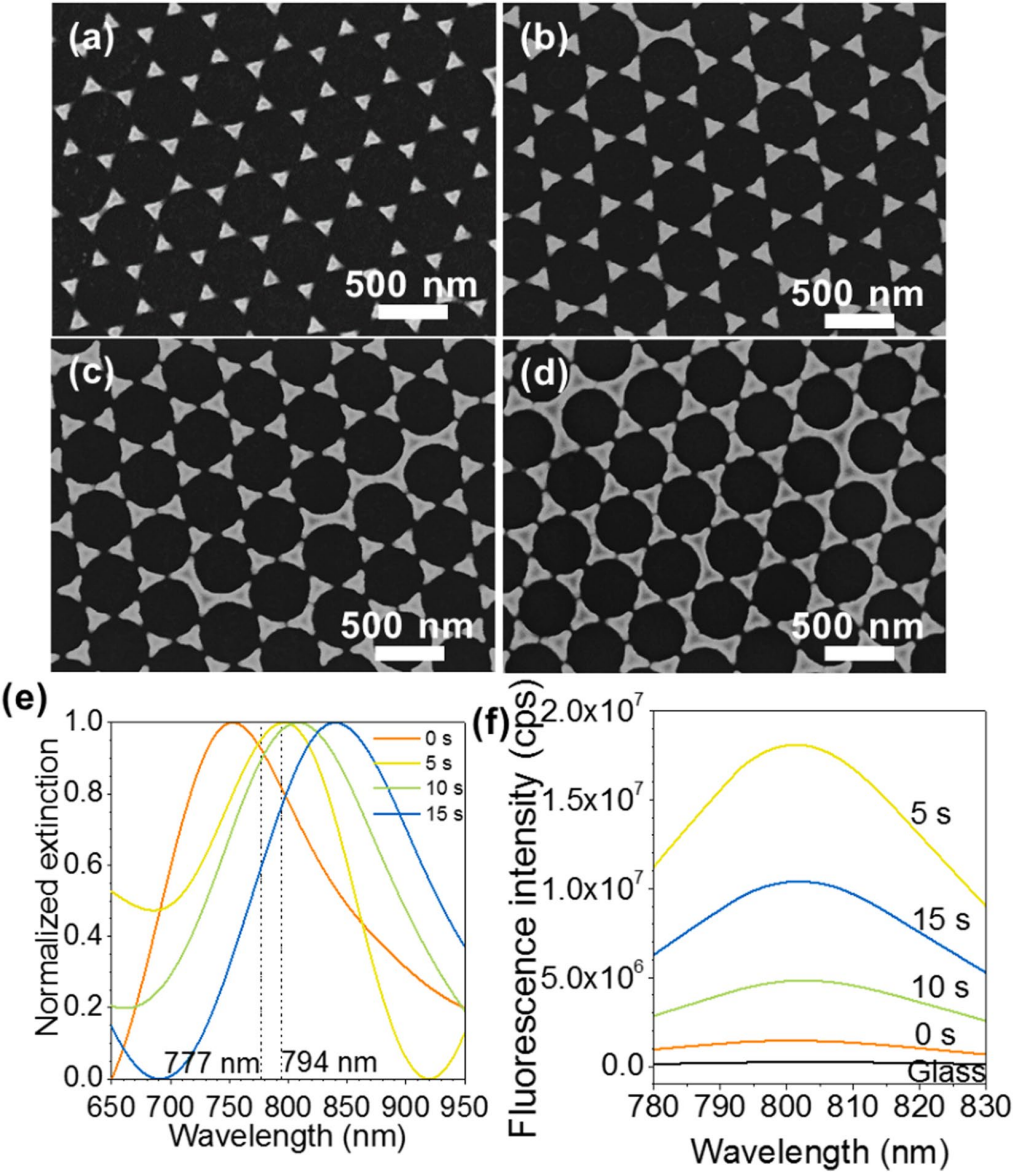
Tunable NIR enhancement factors provided by nanoscale controllable substrate synthesis. (**a**–**d**) Scanning electron microscopy images (scale bars, 500 nm) of nanotriangles formed on glass substrates by colloidal lithography, showing tunable morphology with increasing etching time of the polystyrene sphere template: (**a**) 0 s, (**b**) 5 s, (**c**) 10 s and (**d**) 15 s. (**e**) Normalized extinction spectra of the nanotriangular-like Au arrays with increasing etching time. The dashed lines represent the excitation (777 nm) and emission (794 nm) wavelengths of the NIR dye DyLight 800. (**f**) Fluorescence emission spectra of a sandwich immunoassay for the detection of CA 19–9, performed on the plasmonic Au arrays and bare glass, as a control, using 15.4 U∙mL^−1^ of CA 19–9.

Recent work on plasmonic substrates for protein microarrays has been based on porous Au films formed by dealloying, for which systematic tuning of the localized surface plasmon resonance (LSPR) was not feasible. In the present work, the use of colloidal lithography allowed the fabrication of arrays with nanoscale controlled structure, that were reproducible and tunable. SEM imaging of the nanostructured arrays (Fig. 2a–d) showed that they consisted of ordered Au nanotriangular-like particles, arranged with the original hexagonal close packed structure of the polystyrene (PS) sphere template. Increasing O_2_ plasma etching time of the PS sphere monolayer reduced the size of the spheres, thereby increasing the size of the Au nanotriangles, while reducing the inter-particle separation. The structural characteristics of the Au nanotriangular arrays are summarized in Table 1. Their height, controlled by the e-beam evaporation system, was 100 nm. The use of colloidal lithography has distinct advantages, as it is low cost and allows for large area (in the order of cm^2^) uniform MEF arrays. The combination of colloidal lithography with O_2_ plasma etching, allowed us to tune the properties of the final nanostructures, and control their size and separation distance.

**Table 1:** Structural parameters and extinction peak positions of Au nanotriangular arrays, with the enhancement factors of the NIR dye DyLight 800, measured with each array.

Etching time (s)	*α* (nm)	*s* (nm)	Extinction Peak (nm)	Enhancement factor (E_f_)
0	134 ± 15	98 ± 15	745	12
5	164 ± 18	60 ± 10	796	82.5
10	179 ± 14	46 ± 5	805	29.9
15	186 ± 13	23 ± 10	846	36.5

*α* is the in-plane width (tip-to-tip distance; Supplementary Figure S1) of Au nanoparticles, and *s* is the interparticle distance.

### Au nanostructured arrays show tunable optical properties

The normalized extinction spectra of the Au nanotriangular arrays (Fig. 2e) presented localized surface plasmon resonance (LSPR) peaks in the NIR region. The wavelength of the LSPR peak position (Table 1) increased with increasing etching time. In line with Mie theory^28^, an increase in the size of nanotriangles and reduction in inter-particle separation led to a red-shift in the LSPR peaks. Similar observations have been reported for our work on arrays of silver (Ag) nanoparticles^29^. Therefore, by manipulating their structural characteristics under nanoscale control, the arrays presented optical tunability in the NIR region. This tunability could be valuable for enhancing the performance of protein microarray detection assays, and also allow tunability of their sensitivity parameters.

### CA 19–9 protein microarrays on Au nanotriangular substrates allow up to two orders of magnitude fluorescence enhancement

The possibility to apply the plasmonic properties of our Au nanostructured arrays for increasing the sensitivity of protein microarrays was examined by performing a sandwich assay for the detection of CA 19–9, using an NIR dye (DyLight 800; excitation 777 nm, emission 794 nm). A schematic representation of the antibody assay is shown in Fig. 1. The fluorescence measurements for the sandwich assay performed on Au nanostructured arrays, as well as bare glass substrates used as a control, are shown in Fig. 2f. CA 19–9 was detectable at 15.4 U.mL^−1^ using the Au nanostructured arrays, which afforded a relative fluorescence enhancement of DyLight 800 compared with microarrays on glass substrates. The averaged fluorescence enhancement factors (E_f_) were calculated as previously described^29^, using Equation (1):

1 $${E}_{f}=\frac{{E}_{Au/DyLight800}-{E}_{glass}}{{E}_{glass/DyLight800}-{E}_{glass}}\cdot \frac{{S}_{uncovered}}{{S}_{total}}$$

and are shown in Table 1. The maximum fluorescence enhancement was 82.5, almost two orders of magnitude.

The fluorescence enhancement observed on the Au nanotriangular arrays could be attributed to two possible mechanisms. Firstly, the increase in the local electromagnetic field close to the Au nanostructures, which produces a higher excitation rate for the fluorophores (*i.e*. excitation enhancement). Secondly, fluorophores in proximity to metal nanostructures experience an increase in their radiative decay rate, altering both their fluorescence lifetime and quantum yield, which is termed emission enhancement. In our results, the highest fluorescence enhancement was observed for the sample synthesized with a 5 s etching time. This is due to the higher degree of spectral overlap between the LSPR of this sample with the PL emission spectrum of DyLight 800, which is key to obtaining the maximum fluorescence enhancement^21^.

A higher enhancement factor measured for the sample fabricated with a 15 s etching time, compared to the one with a 10 s etching time, was observed, despite a lower degree of spectral overlap. This is because spectral overlap is only one of several parameters contributing to MEF. Experimental and theoretical results suggest that MEF depends on nanoparticle shape and size, their separation distance and arrangement geometry, the surrounding dielectric medium, as well as the distance between nanoparticles and fluorophores^21^. The electromagnetic field that is thought to be responsible for this enhancement has been modelled in previous work from our group^30^. Using finite-difference time-domain (FDTD) calculations, we have previously shown that the electric field enhancement (in the gap between gold nanotriangles, as well as other regions not occupied by gold) increased as the separation distance between nanotriangles decreased from 100 to 70 and 20 nm^30^. Consequently, the lower inter-particle distance for the sample with a 10 s etching time may have led to a higher excitation enhancement of the fluorophore, leading to a higher enhancement factor. It is also feasible that some fluorophores could couple to more than one metal particle in tightly packed arrays, enhancing the emission further. Fluorophore coupling to the scattering component of LSPR can enhance radiative decay rates, leading to fluorescence enhancement, whereas coupling to the absorption component can enhance non-radiative decay rates, causing fluorescence quenching. In our results, the scattering efficiency of the Au triangular arrays is large in the NIR, overlapping with the fluorescence excitation/emission wavelengths of the DyLight 800 dye.

To obtain further insights into fluorescence enhancement with our nanostructured arrays, we measured the excited state lifetime of DyLight 800. The quantum yield of a fluorophore is defined as the ratio of the radiative decay rate to the total excited state decay rate, which is the sum of the radiative and non-radiative decay rates. A reduction in lifetime was observed, from 880 ps in free space, to 58 ps on the Au nanotriangular platform with 5 s etching time (Supplementary Figure S2), suggesting that a higher radiative decay, caused by fluorophore coupling to the dipolar plasmonic modes in the nanotriangle structure also contributed to the measured increase in enhancement.

### Metal enhanced fluorescence affords increased sensitivity and broadened dynamic range for the detection of CA 19–9

**Figure 3:**
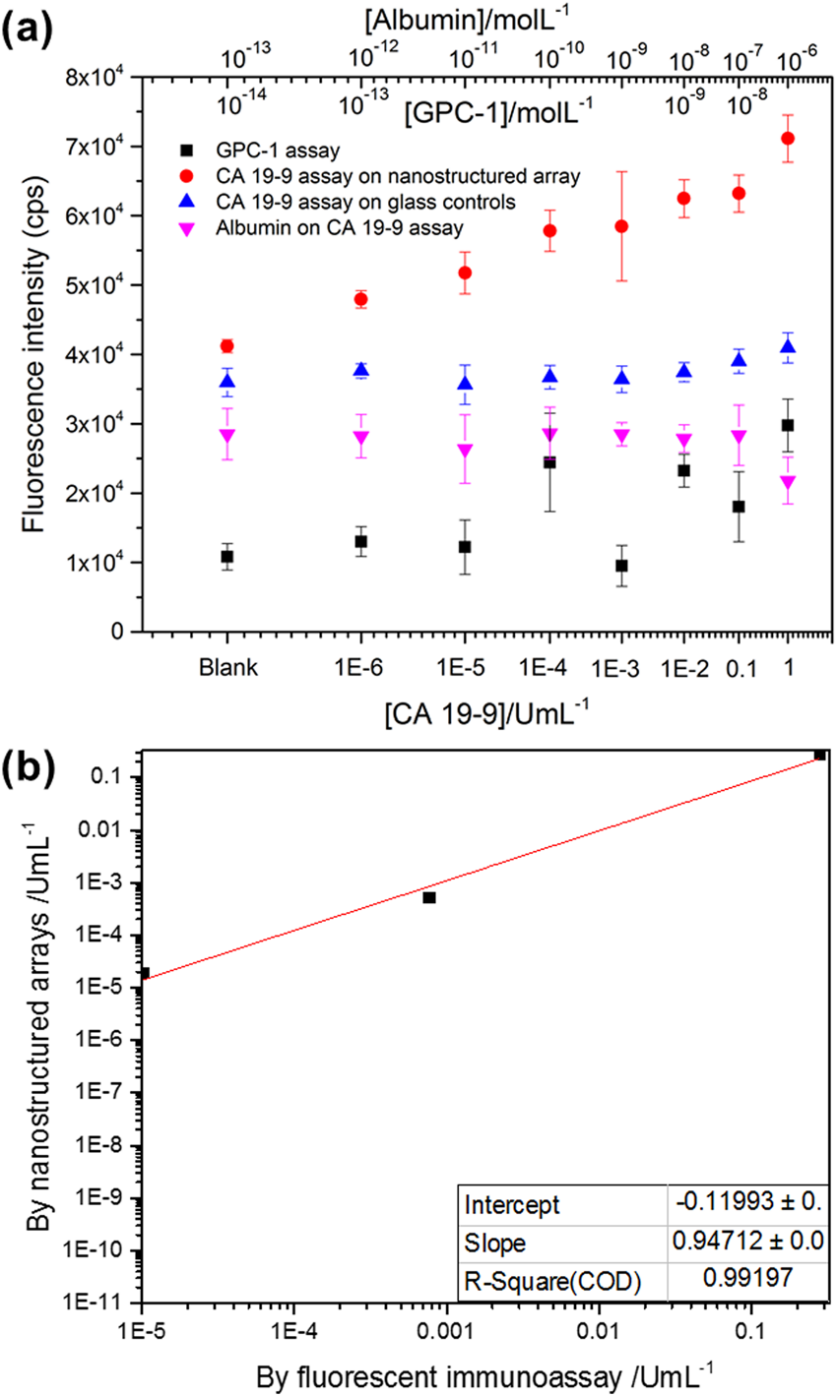
Metal enhanced fluorescence leads to broadened dynamic range and low detection limit for CA 19–9 immunoassays. (**a**) Calibration curve for CA 19–9 quantification, generated by averaging the integrated fluorescence intensity of donkey anti-rabbit Dylight800 emission, over 4 microarray spots, for each CA 19–9 concentration. Error bars represent the standard deviation of the 4 measurements. Similarly, calibration curves for non-specific biomarkers, including BSA and GPC-1 were carried out, showing non-specific binding between BSA/GPC-1 and antibodies. (**b**) Correlation of the plasmonic assay with a standard ELISA immunoassay.

Since the Au arrays fabricated with O_2_ plasma etching times of 5 s led to the highest fluorescence enhancement, these arrays were used to test for the possible improvement in CA 19–9 detection sensitivity, when compared to assays on glass substrates. Identical assays were performed on the Au nanostructured arrays with 5 s etching time, as well as glass substrates, using a serial dilution of CA 19–9 in PBS, to form calibration curves for the quantification of CA 19–9. The fluorescence intensity of donkey anti-rabbit DyLight800 emission was averaged over 4 microarray spots for each CA 19–9 concentration. The calibration curves for CA 19–9 quantification (Fig. 3a), show that the metal enhanced fluorescence afforded by the Au nanostructured array, led to an ultralow detection limit and a broad dynamic range for the detection of CA 19–9. The detection limit, calculated at 3 times the standard deviation of the background signal, was 7.7 × 10^–7^ U.mL^−1^ on Au nanotriangular arrays (R^2^ = 0.966). In contrast, the detection limit of CA 19–9 on the glass substrate control was 0.004 U.mL^−1^. We note that in some cases the error bars are broad, and this may be a result of variation in the washing technique, leading to variation in the background signal, which is inherent to immunoassays performed manually. This variation could be overcome in future by adapting the assay and automating the washing steps. These results demonstrate that the up to two orders of magnitude fluorescence enhancement afforded by Au triangular arrays, dramatically improved the sensitivity of CA 19–9 detection by ~10,000-fold relative to the glass substrate and afforded a broadened dynamic range of six orders of magnitude, as shown in Fig. 3a. The sensitivity offered by the Au nanotriangular arrays is significantly lower than the lower limit of 35 U.mL^−1^ used in current clinical setting. One advantage of this increased sensitivity is that ultralow amounts of sample would need to be collected from patients for testing. The much lower detection limit offered by our assay would allow higher dilutions of such samples, without affecting the ability of our assay to accurately quantify the concentration of CA 19–9.

To validate the specificity of the plasmonic assay based on the Au nanotriangular arrays, the system was challenged against an unrelated globular protein, bovine serum albumin (BSA), as well as another protein, glypican-1 (GPC-1), using various protein concentrations. GPC-1 has recently been identified as another biomarker whose levels may increase in pancreatic cancer^31^. As shown in Fig. 3a, the control experiments did not yield any significant fluorescence intensities, indicating that the effect of non-specific binding between either BSA or GPC-1 and the assay antibodies was negligible. Therefore, these experiments unequivocally demonstrate that the fluorescence intensity observed in the presence of CA 19–9, was not generated by non-specific interactions with the substrate but was in fact related to biospecific recognition by antibodies. Furthermore, this detection limit is far superior to that of commercial immunoassays currently available, including the Elecsys (Roche, Mannheim, Germany) and Architect (Abbott Laboratories, IL, USA)^32^. The limit of quantification calculated at 10 times the standard deviation of the background signal was 2 × 10^−5^ U.mL^−1^. While the precision and reproducibility of the assay need to be determined, its very low detection limit represents a great improvement to other assays for CA 19–9, that display detection limits in the mU.mL^−1^ range^33^. Another novel feature of the array presented here, is the ability to produce substrates with tunable enhancement factors for varied sensitivity, which has not been previously reported, but would be a significant step forward in the development of multiplexed detection platforms.

### Validation in serum samples

Having confirmed that the nanoplasmonic assay was able to detect a broad range of concentrations of CA 19–9, we proceeded to test its performance *in vitro*, using donkey serum samples spiked with CA 19–9 standards at 3 different concentrations spanning the dynamic range of the assay. The detected fluorescence intensities for these samples were compared with the calibration curve to extract the CA 19–9 concentration measured in serum (Fig. 3b). As a comparison, a standard ELISA immunoassay was also used to measure the CA 19–9 level in these samples, which were in agreement with the results obtained using our plasmonic nanosensors. The two methods displayed a positive correlation with a slope of 0.9471, an intercept of −0.1199 and a correlation co-efficient of 0.9920 (Fig. 3b). The detection of CA 19–9 in donkey serum demonstrated the robustness of these plasmonic nanosensors against interferences in a complex matrix. Therefore, these findings show promise in clinical applications, paving the way for pin-prick blood tests for cancer diagnosis and post-surgery monitoring. Along with ongoing efforts by us and other groups to combine CA19–9 with other biomarkers in order to improve the accuracy of diagnosis^27^, our proof-of-concept study using CA 19–9 could easily be extended to use any other biomarker of interest, which would further advance the field of cancer diagnostics and early detection.

## Conclusions

In summary, we have demonstrated tunable fluorescence enhancement of protein microarrays in the NIR region using nanostructures produced by colloidal lithography. The plasmonic arrays offered a much wider dynamic range and lower detection limit compared to glass controls, owing to the improvement in signal-to-noise ratio, without complicating the measurement procedure. The nanoplasmonic assays were validated in donkey serum samples. The substrates are fully compatible with microarray scanners in current use. Along with the improvement in sensitivity, and tunability of this sensitivity, this opens up the possibility of using the protein microarray approach to detect a range of biomarkers in a small sample volume for the early diagnosis of pancreatic cancer. Further work is underway to optimize the assay on different substrates, to extend the range of sensitivities, and apply the assay to other proteins in a multiplexed approach.

## Methods

### Materials

Polystyrene microspheres with diameter of 400 nm (10 wt. %) were obtained from Bangs Laboratories Inc., USA. Cysteamine, (3-Aminopropyl)triethoxysilane (APTES), 4arm-Polyethylene glycol-Carboxyl (Mn 10,000), dimethylformamide (DMF), 1-ethyl-3-[3-(dimethylamino)propyl]-carbodiimide hydrochloride (EDC), N-Hydroxysuccinimide (NHS), donkey serum, Dylight800 conjugated polyclonal donkey anti-rabbit antibody and phosphate buffered saline (PBS, pH 7.4) were purchased from Sigma-Aldrich, UK. Nanopure water (>18.2 MΩ), purified using the Millipore Milli-Q gradient system, was used in all the experiments. Glass microscope slides were obtained from VWR International, USA and rinsed with acetone, 2-propranol and nanopure water before use. Purified Carbohydrate antigen (CA 19–9) and monoclonal mouse anti-CA 19–9 antibody were purchased from Biospacific. Polyclonal rabbit anti-CA 19–9 antibody was purchased from Lifespan Biosciences.

### Fabrication of nanotriangular arrays

The ordered Au nanotriangular arrays were produced by a modified colloidal lithography allowing nanoscale control of size, shape, and inter-particle separation. First, templates of polystyrene (PS) sphere monolayers were fabricated through an adapted colloidal lithography technique, as previously reported^29^. Briefly, 20 μL of a polystyrene sphere (400 nm diameter) solution in ethanol was applied to a clean silicon wafer, which was subsequently submerged slowly in a glass container filled with deionized water. The PS particles formed a monolayer on the water surface which was lifted using clean glass substrates. Once the templates were formed, the arrays were etched for 0, 5, 10 or 15 seconds using O_2_ plasma, and coated with a Au layer with a thickness of 100 nm using a Mantis e-beam evaporation system. Following PS template removal, the morphology of the Au nanostructured arrays was examined by scanning electron microscopy (SEM) using a LEO Gemini 1525 field emission gun SEM. The optical properties of the Au nanostructured arrays were measured using a Cary 5000 UV-Vis-NIR spectrophotometer.

### CA 19–9 fluorescent immunoassay

To prepare protein microarrays on the Au nanostructured films, the array surfaces were first modified with branched poly(ethylene glycol)-carboxylate (PEG-carboxylate), through covalent conjugation to an amine self-assembled monolayer. This amine monolayer was formed by serially incubating the Au nanostructured arrays with cysteamine and APTES, to ensure coverage of both glass and Au surfaces. The NHS-activated substrates were incubated with monoclonal mouse anti-CA 19–9 immunoglobulin G (IgG) in phosphate buffered saline (PBS). The substrates were then blocked overnight at 4 °C. CA 19–9 was diluted into PBS at a concentration of 15.4 U.mL-1 and this solution was applied to the substrates. The substrates were then incubated with polyclonal rabbit anti-CA 19–9 antibody followed by washing and incubation with DyLight 800-conjugated donkey anti-rabbit antibody.

### Fluorescence measurement and analysis

The fluorescence emission spectra were collected using a Fluorolog Tau 3 system from Horiba Jobin Yvon with a 450 W Xenon lamp excitation. The samples were excited at 755 nm, and their fluorescence was measured in the range of 780–830 nm using 5 nm slits. The mean and standard deviation over four measurements were taken. Fluorescence decay curves of Dylight800 conjugated sandwich assays on nanotriangle substrates and glass controls were measured by time-correlated single photon counting (TCSPC) using a 723 nm picosecond pulsed diode laser.

### Specificity of fluorescent immunoassay

To evaluate the specificity of the fluorescent immunoassay, bovine serum albumin (BSA) at concentrations between 10^−6^ and 10^−13^ mol.L^−1^, and glypican-1 (GPC-1) at concentrations between 10^−7^ mol.L^−1^ and 10^−14^ mol.L^−1^, were prepared by diluting BSA or GPC-1 in PBS. Each dilution was applied to the fluorescent immunoassay to determine the specificity.

### Validation in serum samples

To assess the potential *in-vitro* performance of the assay, for the detection of CA 19–9 at concentrations spanning the full dynamic range, samples were prepared by spiking CA 19–9 standards at different concentrations into donkey serum. The CA 19–9 content of the serum samples was measured using the plasmonic protein microarrays, as described in the section “CA 19–9 fluorescent immunoassay”, and compared to a standard ELISA fluorescent immunoassay.

## Electronic supplementary material

Supplementary Information
