# Supplementary material for: Highly Sensitive Plasmonic Detection of the Pancreatic Cancer Biomarker CA 19-9
Source: Sci Rep. 2017 Oct 30;7:14309. doi: 10.1038/s41598-017-14688-z (PMC5662715; doi:10.1038/s41598-017-14688-z)
Supplement: Supplementary file 1 — Supplementary Information [file 41598_2017_14688_MOESM1_ESM.pdf]

Supplementary Information

Highly Sensitive Plasmonic Detection of the Pancreatic Cancer Biomarker CA 19-9

Zaynab A. R. Jawad[a,b], Ioannis G. Theodorou[a], Long R. Jiao[b] & Fang Xie[a,*]

[a]Department of Materials and London Centre for Nanotechnology, Imperial College London, SW7 2AZ London, UK

[b]Department of Cancer and Surgery, Imperial College London, SW12 0HS London, UK

*f.xie@imperial.ac.uk

ABSTRACT: Plasmonic gold (Au) nanotriangular arrays, functionalized with a near infrared (NIR) fluorophore-conjugated immunoassay to Carbohydrate Antigen 19-9 (CA 19-9), a pancreatic cancer biomarker, produce optically tunable substrates with two orders of magnitude fluorescence enhancement. Through nanoscale morphological control, the sensitivities of the plasmonic nanotriangular arrays are controllable, paving the way of such optical platforms for multiplexing. Here, we report a limit of detection (LOD) of 7.7×10^{-7} U.mL^{-1} for CA 19-9 by using such tunable Au nanotriangular arrays, a great improvement compared to commercially available CA 19-9 immunoassays. The linear dynamic range was from 1×10^{-6} U.mL^{-1} to 1 U.mL^{-1}, *i.e.* up to six orders of magnitude. Moreover, high specificity was demonstrated, together with successful validation in serum samples. Their superior tunable sensitivity, along with efforts to combine CA 19-9 with other biomarkers for improved accuracy, open up the possibility for multiplexed NIR-fluorescence enhancement microarrays, for early cancer diagnosis and therapeutic monitoring.

Materials:

Polystyrene microspheres with diameter of 400 nm (10 wt. %) were obtained from Bangs Laboratories Inc., USA. Cysteamine, (3-Aminopropyl)triethoxysilane (APTES), 4arm-Polyethylene glycol-Carboxyl (Mn 10,000), dimethylformamide (DMF), 1-ethyl-3-[3-(dimethylamino)propyl]-carbodiimide hydrochloride (EDC), N-Hydroxysuccinimide (NHS), donkey serum, Dylight800 conjugated polyclonal donkey anti-rabbit antibody and phosphate buffered saline (PBS, pH 7.4) were purchased from Sigma-Aldrich, UK. Nanopure water (>18.2 MΩ), purified using the Millipore Milli-Q gradient system, was used in all the experiments. Glass microscope slides were obtained from VWR International, USA and rinsed with acetone, 2-propranol and nanopure water before use. Purified Carbohydrate antigen (Ca19-9) and monoclonal mouse anti-Ca19-9 antibody were purchased from Biospacific. Polyclonal rabbit anti-Ca19-9 antibody was purchased from Lifespan Biosciences.

Methods:

Surface modification of nanostructured arrays:

To prepare protein microarrays on the Au nanostructured films, the array surfaces were first modified with branched poly(ethylene glycol)-carboxylate (PEG-carboxylate), through covalent conjugation to an amine self-assembled monolayer. This amine monolayer was formed by serially incubating the Au nanostructured arrays with cysteamine and APTES, to ensure coverage of both glass and Au surfaces.

CA19-9 sandwich assay procedure:

Polyclonal mouse anti-Ca19-9 antibody was applied at a concentration of 5uM to the substrates and incubated for 2 hours on an orbital shaker. Following this the substrates were blocked overnight at 4°C in 3% donkey serum in PBS containing 0.05% tween-20 (PBST) supplemented with 1 mM tris. Purified carbohydrate antigen 19-9 (Ca19-9) was diluted into PBS and 40 uL was applied to the substrates,

together with a control of PBS only (blank). The substrates were incubated at room temperature in a humidified atmosphere for six hours on an orbital shaker (100RPM) followed by removal of the Ca19-9 by micropipette, two immersions on PBST (five minutes each) and a brief immersion in PBS. Polyclonal rabbit anti-Ca19-9 was applied to each substrate at 3 x 10-7 M and incubated for 1.5 hours at room temperature on an orbital shaker. Assays were washed as above. 1.6×10^{-8} M of Dylight800 conjugated polyclonal Donkey anti-Rabbit IgG was incubated at room temperature on each substrate for 10 minutes. Substrates were washed x3 in PBST and x1 in PBS, followed by rinsing in nanopure water.

Preparation of Au nanostructured arrays:

Monodisperse polystyrene microspheres with diameter of 400 nm were diluted with ethanol at a 1:1 ratio. About 3-5 μL of the above PS solutions were placed onto the surface of a clean silicon wafer (~ 30 mm × 20 mm), which had been kept for 24 h in 10% sodium dodecyl sulfate. The wafer was subsequently submerged slowly in a 15 cm glass container filled with 150 mL of nanopure water, causing the PS spheres to form a disordered monolayer on the water surface. Addition of 4 μL of 2% sodium dodecyl sulfate, changed the surface tension of the water and allowed a monolayer with highly ordered areas to be obtained. The PS monolayers were collected from the water surface using clean glass substrates. Following formation of the PS sphere templates, a Mantis e-beam evaporation system fitted with a deposition monitor quartz crystal microbalance was used for the deposition of an Au layer, with a thickness of 100 nm or 50 nm. The PS sphere template was removed by sonicating the substrates in absolute ethanol for 2 min.

The Au nanostructures were imaged by scanning electron microscopy (SEM) using a LEO Gemini 1525 field emission gun (FEG) SEM (Carl Zeiss Microscopy GmbH, UK). The SEM was operated in secondary electron mode at an accelerating voltage of 5 kV, using the InLens detector. The optical

properties of the Au nanostructured arrays were measured at room temperature using a Cary 5000 UV-Vis-NIR spectrophotometer.

Fluorescence measurement and analysis:

The fluorescence emission spectra were collected using a Fluorolog Tau 3 system from Horiba Jobin Yvon with a 450 W Xenon lamp excitation. The samples were excited at 755 nm, and their fluorescence was measured in the range of 780–830 nm using 5 nm slits. The mean and standard deviation over four measurements were taken. Fluorescence decay curves of Dylight800 conjugated sandwich assays on nanotriangle substrates and glass controls were measured by time-correlated single photon counting (TCSPC) using a 723 nm picosecond pulsed diode laser.

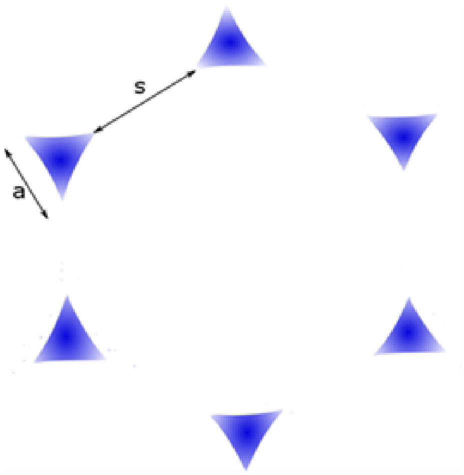

Figure S1. Dimensions of nanotriangles synthesised; a, is tip-to-tip distance, s, is interparticle distance.

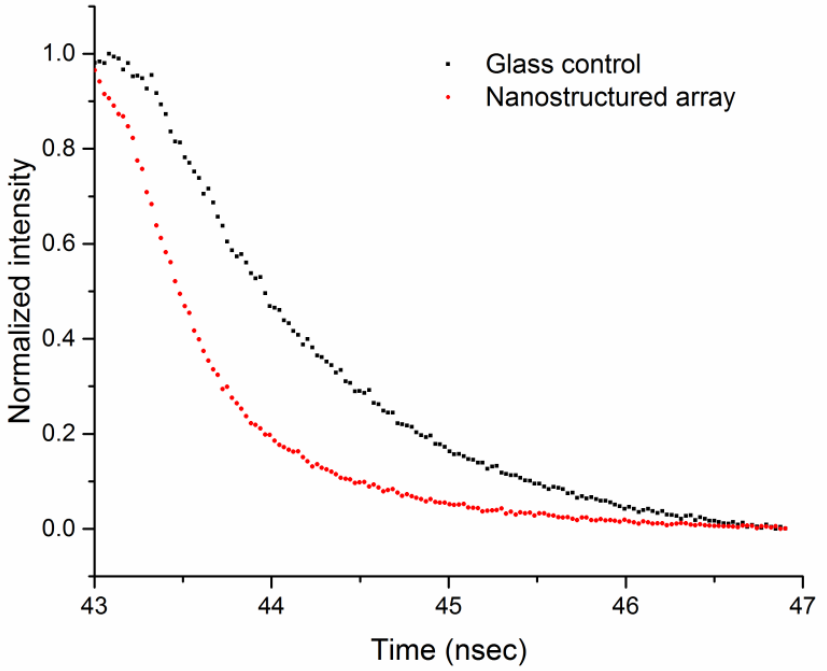

Figure S2. Fluorescence lifetime measurements of nanotriangle substrates compared to glass.
